# Influence of the Fly Ash Material Inoculants on the Tensile and Impact Characteristics of the Aluminum AA 5083/7.5SiC Composites

**DOI:** 10.3390/ma14092452

**Published:** 2021-05-09

**Authors:** Santhosh Nagaraja, Kempaiah Ujjaini Nagegowda, Anand Kumar V, Sagr Alamri, Asif Afzal, Deepak Thakur, Abdul Razak Kaladgi, Satyam Panchal, Ahamed Saleel C

**Affiliations:** 1 Department of Mechanical Engineering, MVJ College of Engineering, Bengaluru 560067, India; 2 Department of Mechanical Engineering, University Visvesvaraya College of Engineering, Bangalore University, Bengaluru 560001, India; unkempaiah@bub.ernet.in; 3 S.E.A College of Engineering and Technology, Bengaluru 560049, India; aanandrv@gmail.com; 4 Department of Mechanical Engineering, College of Engineering, King Khalid University, P.O. Box 394, Abha 61421, Saudi Arabia; salamri@kku.edu.sa (S.A.); ahamedsaleel@gmail.com (A.S.C.); 5 Department of Mechanical Engineering, P.A. College of Engineering, Karnataka 574153, India; arkmech9@gmail.com; 6 Chitkara University Institute of Engineering and Technology, Chitkara University, Punjab 140401, India; deepak.thakur@chitkara.edu.in; 7 Department of Mechanical and Mechatronics Engineering, University of Waterloo, Waterloo, ON N2L 3G1, Canada; satyam.panchal@uwaterloo.ca

**Keywords:** fly ash, material inoculants, interatomic, grain growth, aluminum, composites

## Abstract

The choice of suitable inoculants in the grain refinement process and subsequent enhancement of the characteristics of the composites developed is an important materials research topic, having wide scope. In this regard, the present work is aimed at finding the appropriate composition and size of fly ash as inoculants for grain refinement of the aluminum AA 5083 composites. Fly ash particles, which are by products of the combustion process in thermal power plants, contributing to the large-scale pollution and landfills can be effectively utilized as inoculants and interatomic lubricants in the composite matrix–reinforcement subspaces synthesized in the inert atmosphere using ultrasonic assisted stir casting setup. Thus, the work involves the study of the influence of percentage and size of the fly ash dispersions on the tensile and impact strength characteristics of the aluminum AA 5083/7.5SiC composites. The C type of fly ash with the particle size in the series of 40–75 µm, 76–100 µm, and 101–125 µm and weight % in the series of 0.5, 1, 1.5, 2, and 2.5 are selected for the work. The influence of fly ash as distinct material inoculants for the grain refinement has worked out well with the increase in the ultimate tensile strength, yield strength, and impact strength of the composites, with the fly ash as material inoculants up to 2 wt. % beyond which the tensile and impact characteristics decrease due to the micro coring and segregation. This is evident from the microstructural observations for the composite specimens. Moreover, the role of fly ash as material inoculants is distinctly identified with the X-Ray Diffraction (XRD) for the phase and grain growth epitaxy and the Energy Dispersive Spectroscopy (EDS) for analyzing the characteristic X-Rays of the fly ash particles as inoculant agents in the energy spectrum.

## 1. Introduction

Aluminum composites have gained significant use in the past decade, owing to their greater strength characteristics and better mechanical properties leading to optimization in design and development of components for automobile, aerospace and marine applications. The potential for newer reinforcements for the aluminum composites is of greater significance, particularly with respect to the sustainability and material savings [1]. Metal matrix composites have triggered explicit interest especially among materials scientists for the synthesis of defect free high strength composites; this is possible with the use of suitable material inoculants, which improve modulus, strength, and fatigue and fracture resistance at elevated temperatures [2]. The choice of suitable material inoculants is gaining importance primarily in synthesis of aluminum composites, because of their ability to yield high specific strength, high stiffness, wear resistance, which eventually find their applications in automotive elements like pistons, cylinder liners, brake rotors and connecting rods [3].

The use of SiC and other ceramic reinforcements has their effect on the yield strength, plasticity, and wear of Aluminum composites; however, the entirety of these properties depend on the composition and the distribution of these reinforcements in the matrix phase [4]. The composition of alloying elements like Si, Fe, and Mg plays a significant role in enhancing the mechanical properties of Al/SiC composites [5]. The bonding between these elements is significant for enhancing the characteristics of the composites. In this regard, the inoculants bring about distinct atomic bonding between the elements to enhance the characteristics of the composites [6]. The elastic modulus and the tensile strength characteristics of SiC particulate reinforced composites have exhibited better compressive strength characteristics due to the inclusion of the hard ceramic reinforcements and the SiO_2_ inoculants [7]. In general, the hardness of the matrix phase and compression strength augment with the inclusion of material inoculants, along with the impact strength and the porosity of the composites, limiting the grain growth and structural porosity [8]. Hardness and strength of composite materials are augmented with a rise in the weight percentage of the SiO_2_ inoculants; precipitation hardening causes a further increase in hardness of composite materials [9]. Increase in composition of SiO_2_ inoculants in the metallic matrix augmented the hardness, impact strength, and normalized displacement within the samples synthesized by a two-step stir casting technique [10]. Mechanical properties are determined by the reinforcement content, its distribution, the wettability with the matrix materials, and conjointly the porosity content [11]. Through a correct combination of processing methodology, uniform particle distribution and the appropriate dispersion of the reinforcement particles, the necessary properties shall be obtained [12]. The properties of metal matrix composites are greatly influenced by the distribution of reinforcements in the matrix and the grain size, fibre arm spacing, size and morphology of atomic orientations, etc. [13]. In case of the fracture of composites during tensile test, throughout the process of tensile fracture of the aluminum/SiCp composites, the inoculants can restrict the nucleation of micro cracks once the surface cohesion between the reinforcements and matrix becomes strong; decohesion between the reinforcements and matrix can nucleate micro cracks before the onset of debonding, subject to weakening of the forces between the atoms in the composites [14]. Internal fracture in composites is because of the plastic deformation owing to particle debonding and matrix cracking [15].

Composites reinforced with larger size of SiO_2_ (35–45 μm) material inoculants exhibited higher strength characteristics than composites with smaller SiO_2_ particles (18–23 μm) [16]. The crack initiation and fracture of the coarse particle reinforced composite were principally related to debonding within the matrix around individual particles prior to main crack tip; the boundaries between the particle clusters and closely bonded matrix are liable for the failure in case of inoculants free compositions for the composites [17]. Elimination of residual pores and defects is possible with the controlled process for the synthesis of composites, and the distribution of the reinforcements in these composites is a major aspect that conforms the stronger bonding between the matrix and the reinforcements. The stronger bonding between the matrix and particles and refinement of grains improved the impact strength of composites [18]. The inclusion of material inoculants, especially the SiO_2_, Al_2_O_3,_ has augmented the strength and damping characteristics of the metal matrix composites, particularly due to the coring and anisotropic grain boundary migration in the matrix phase [19,20]. Addition of phenoplasts provides systematically higher coefficients of friction and low wear rates, especially in the aluminum matrix composites [21]. The mechanical properties and wear resistance of composites with elongated flakes of fly ash exhibited superior properties as compared to thicker stubs of fly ash particles [22,23]. Rohatgi et al. [24] have reported that the inclusion of fly ash reinforcements up to 20 wt. % can conserve up to 39 kWh energy for each kg of aluminum composites cast; further, the replacement of aluminum up to 10 wt. % can result in conservation of 93 kWh power requirements, especially in automobiles and aerospace industry. Kasar, A.K., et al. [25] have reviewed the use of Fly Ash in polymer and metal matrices resulting in enhanced mechanical and tribological characteristics especially for industrial applications ranging from automotive to aerospace components with multi utility approach. Anil Kumar et al. [26] have investigated the mechanical characteristics of AA 6061 reinforced with Fly Ash particles of varying size and composition and have reported that the addition of fly ash particles increases the tensile strength and hardness, which is the primary mandate, especially for the use of composites for aerospace applications. Juang et al. [27] have worked on the friction stir processing of aluminum composites with cenospheres and have reported that the stirring action has resulted in crushing the cenospheres, thereby reducing the particle size and facilitating the uniform distribution of censospheres in the matrix. With this purview, the present investigation is aimed at characterizing the effect of composition and particle size of fly ash as inoculants on the tensile and impact characteristics of the aluminum AA 5083 alloy known for its high strength, anti-oxidative properties, especially with respect to its use in extreme environments, reinforced with 7.5 wt. % SiC ceramic particulates known for its hardness characteristics.

## 2. Materials and Methods

**Figure 1:**
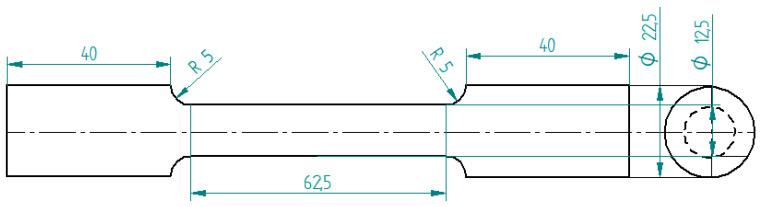
Schematic of Tensile test specimen (unit: mm).

**Figure 2:**
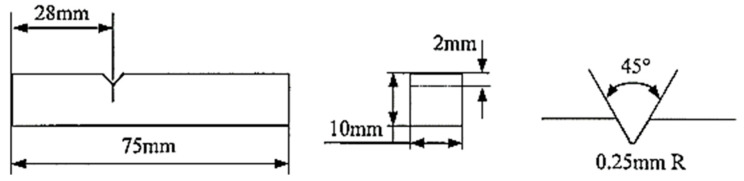
Schematic of Izod test specimen.

Fly ash of Class C type obtained from the combustion of lignite and sub bituminous form of coal at the Raichur Thermal Power Station (RTPS) located in Raichur, India, was initially subjected to sieving and three size ranges were appropriated from the process by considering the size ranges of 40–75 μm, 76–100 μm, and 101–125 μm based on the grain fineness index of the vibratory sieve setup, subsequently followed by the identification of the minima and maxima for the size range of the fly ash particles sourced, further the size range of the fly ash are fixed by dividing the particles into three sets, viz., smaller granules, mid-range particulates, and larger flakes. The quantities of the fly ash inoculants were fixed at 0.5, 1, 1.5, 2, and 2.5 wt. % based on various factors, viz., wettability phenomena, inoculation (i.e., the minima getting fixed considering the tendency to inoculate the grains), followed by the agglomeration (i.e., the maxima getting fixed considering the threshold limit for the tendency for the agglomeration of fly ash flakes to occur in the matrix). The composition of the fly ash particles was considered to be close to the C type from the chemical analysis (Table 1) and comparisons with the ASTM (C618) standards. The fly ash flakes were initially preheated for 10 min to remove the moisture content before adding to the molten pool of aluminum AA 5083 with Mg as the major alloying element (Table 2) reinforced with the SiC along with 0.5 g of Mg to increase the wettability and bonding characteristics of the composites. The stirring was carried out in an inert gas atmosphere and degassed at a temperature of 800 °C using hexacholoroethane (C_2_Cl_6_) tablets, and the slag formed on the top of molten metal was then removed before pouring the melt into the metallic dies (ϕ 23 mm and L 150 mm). The castings of aluminum AA 5083/7.5SiC with the fly ash material inoculants (0.5, 1, 1.5, 2, and 2.5 wt. %) were then machined on a “Kirloskar” make CK50T lathe machine, Bangalore, India, with special attachments to dimensions for tensile (Figure 1) and impact (Izod) tests (Figure 2).

The Fine Instruments make TUE–C model computerized UTM facility at the MVJ College of Engineering, Bangalore, India, was used for carrying out the tensile tests at the ambient conditions in accordance with the ASTM E8-95 standards, by a constantly increasing load and cross head travel of 2 mm/min until the yielding occurred, subsequently followed by sustained loading beyond the plastic zone until the specimen fractured. Three trials were carried out for specimens from each of the composition, and the average values were considered to normalize for any variation during the test conditions. Similarly, Izod impact tests were carried out at the ambient conditions in accordance with the ASTM D 256 standard on an AMT–8 pendulum loading impact machine. Further, the micro structural characterizations were carried out using a Hitachi make SU-8000 series SEM (Tokyo, Japan) available at the University Visvesvaraya College of Engineering, with an accelerating voltage of 20–25 kV at magnifications of 300× to study the influence of fly ash material inoculants on the grain growth epitaxy of the composites and the EDS using silicon drift detector with the detection area of 100 mm^2^ to substantiate the findings by evaluating the presence of inoculant agents, followed by the XRD analysis with the monochromatic Cu Kα radiation in correspondence to the x-ray wavelength of 0.154 nm (λ = 0.154 nm) using a Bruker make machine (Billerica, MA, USA), available at the Indian Institute of Science, for the crystallization and homogenization studies.

## 3. Results and Discussions

The influence of size and composition of the fly ash as inoculants on the tensile and impact characteristics of the AA 5083/7.5SiC composites and the nature of inoculation followed by the grain growth epitaxy are reported and discussed.

### 3.1. Influence of Inoculation of Fly Ash on Tensile Characteristics

The influence of inoculation of fly ash on the tensile characteristics, particularly the size and the weight percentage on the strength and ductility of the composite specimens, are understood from the double Y axis graph.

**Figure 3:**
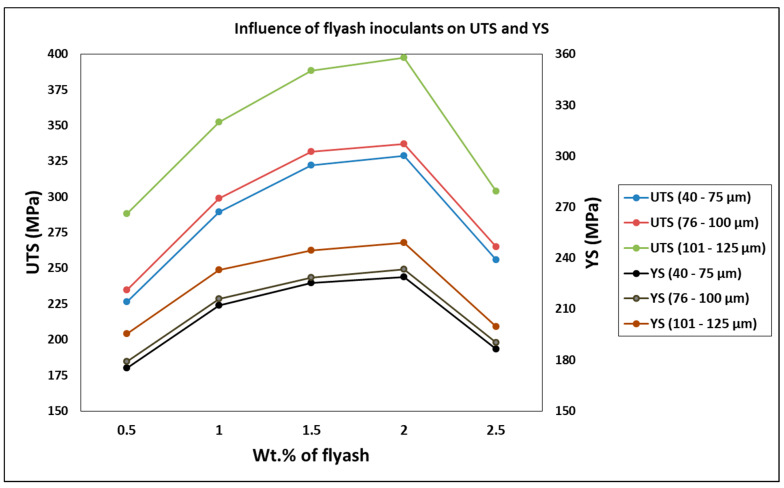
Influence of fly ash inoculants on UTS and YS.

The variation of the Ultimate Tensile Strength (UTS) and the Yield Strength (YS) with the weight percentage and size ranges of fly ash as inoculants is presented graphically in Figure 3.

The UTS and YS of the composites increased steeply with the initial addition of fly ash material inoculants (up to 1 wt. %), subsequently followed by smaller increments in the UTS and YS up to 2 wt. %, attributed to inoculation bringing about grain packing and bonding between the atoms, thereby increasing the strength. However, for 2.5 wt. %, there is a drastic decrease in the ultimate and yield strength due to agglomeration and porosity in the composite, attributed to micro coring and segregation with the creation of voids and crystal imperfections with the increased O-content due to the breaking of bonds between the Si and O_2_ atoms in the fly ash material inoculants.

**Figure 4:**
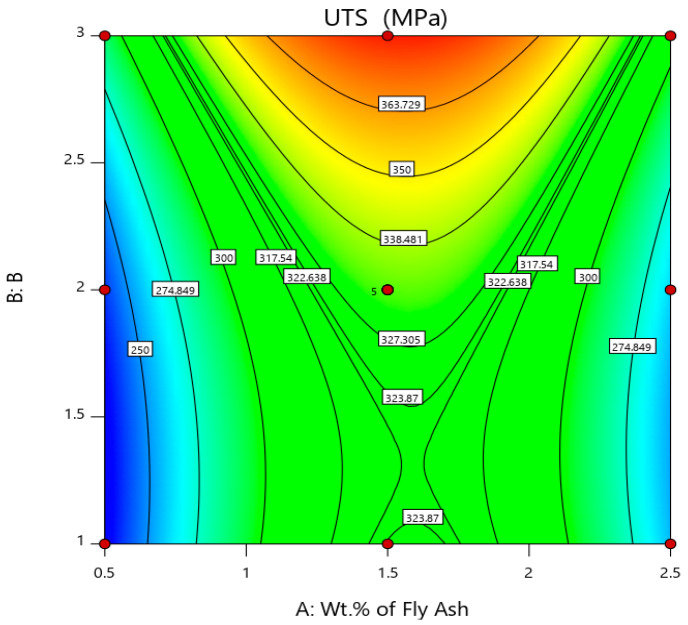

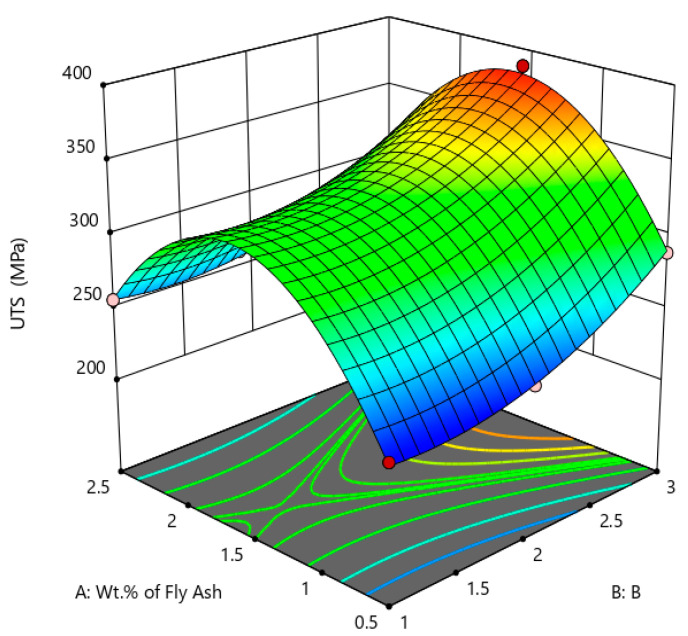
Surface plot and 3D contour plot representing the influence of wt. % and size of the fly ash inoculants on UTS.

**Figure 5:**
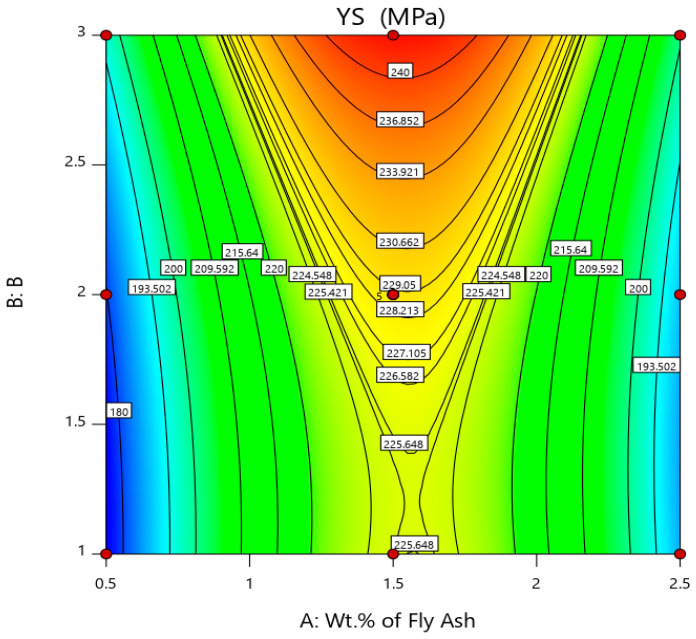

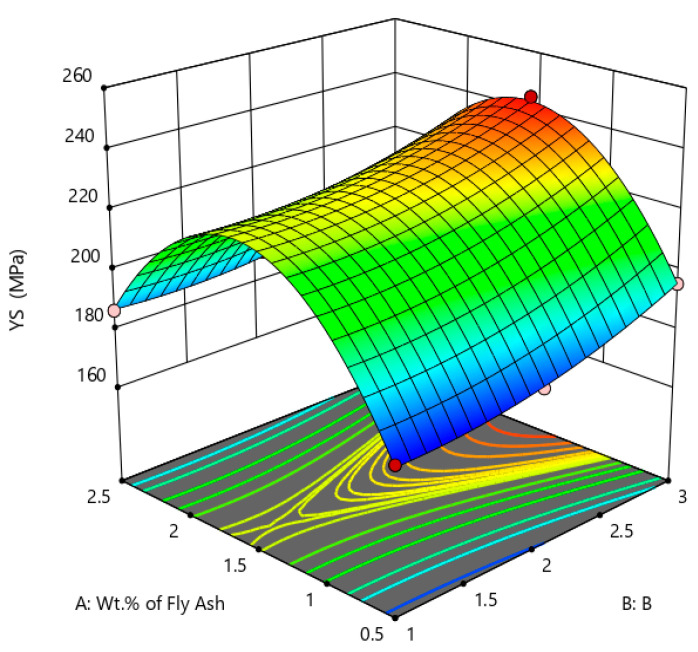
Surface plot and 3D contour plot representing the influence of wt. % and size of fly ash inoculants on YS.

The lowest UTS and YS are observed in AA 5083/7.5SiC with 0.5 wt. % of 40–75 μm fly ash material inoculants, viz., 226.4 MPa and 175.2 MPa, respectively, while the highest UTS and YS are observed in AA 5083/7.5SiC composite with 2 wt. % of 101–125 μm fly ash as material inoculants, viz., 397.6 MPa and 249.2 MPa. Thus, increases in UTS and YS by about 75.61% and 42.23% are observed in composites with 2 wt. % fly ash inoculants. However, beyond 2 wt. % fly ash, the inoculants agglomerate and result in voids and porosities. The increase in UTS and YS with an increase in fly ash material inoculants may be a result of strong atomic packing of inoculants within the AA 5083/7.5SiC composite. Further, the flattened fly ash with 101–125 μm size range exhibits better strength characteristics due to the strong inherent bonding between the interstitial atoms and better packing resulting in greater strength. The surface and 3D contour plots in Figure 4 and Figure 5 give the account of variation of UTS and YS, respectively, with wt. % and size of the fly ash flakes.

The surface and 3D contour plots are plotted considering wt. % of fly ash along A abscissa and size of fly ash (B) coded as 1 for 40–75 μm, 2 for 76–100 μm and 3 for 101–125 μm along B (Z-axis). From, the surface plots and 3D contour plots representing the influence of wt. % and size of the fly ash on the UTS and YS, it is herewith evident that the ultimate tensile strength and yield strength is maximum for 1.5 to 2 wt. % interval of fly ash with size range of 101–125 μm.

The strength characteristics enhance with the inclusion of fly ash particles as inoculants particularly for flakes of fly ash of more than 100 μm. This is also supported by the findings of the microstructural observations and the EDS and the XRD pattern. The findings are in parlance with those reported by some of the researchers who have worked on the use of ceramic particulates as inoculants. Kok et al. [28] have reported about the effect of Al_2_O_3_ particle dispersions in the matrix, the Al_2_O_3_ particles with 32 μm particle size had better strength characteristics as compared to 16 μm particle dispersions in the matrix, which is in line with the findings of the present work, viz., the strength increases with the increase in size of inoculant particles. Kumar et al. [29] have reported the findings related to the influence of fly ash reinforcements on the tensile strength of Al/3Cu/8.5Si and have drawn similar inferences of increase in the strength with the increase in wt. % of fly ash attributed to close packing of the reinforcements in the matrix.

**Figure 6:**
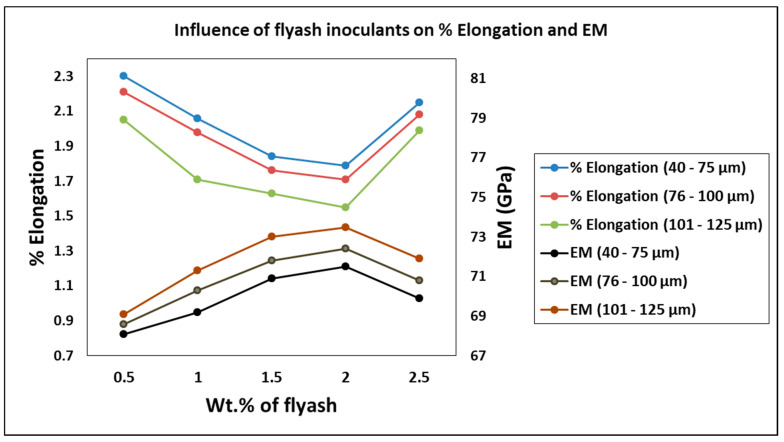
Influence of fly ash inoculants on % Elongation and EM.

**Figure 7:**
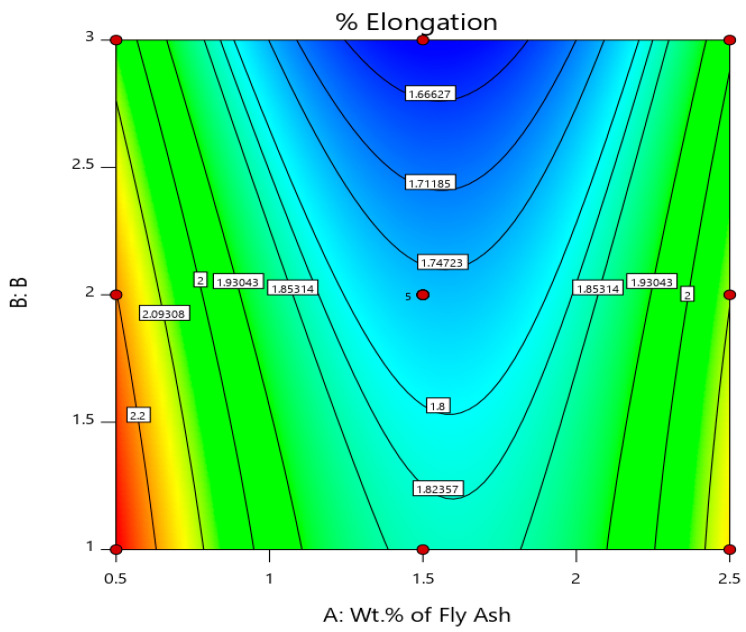

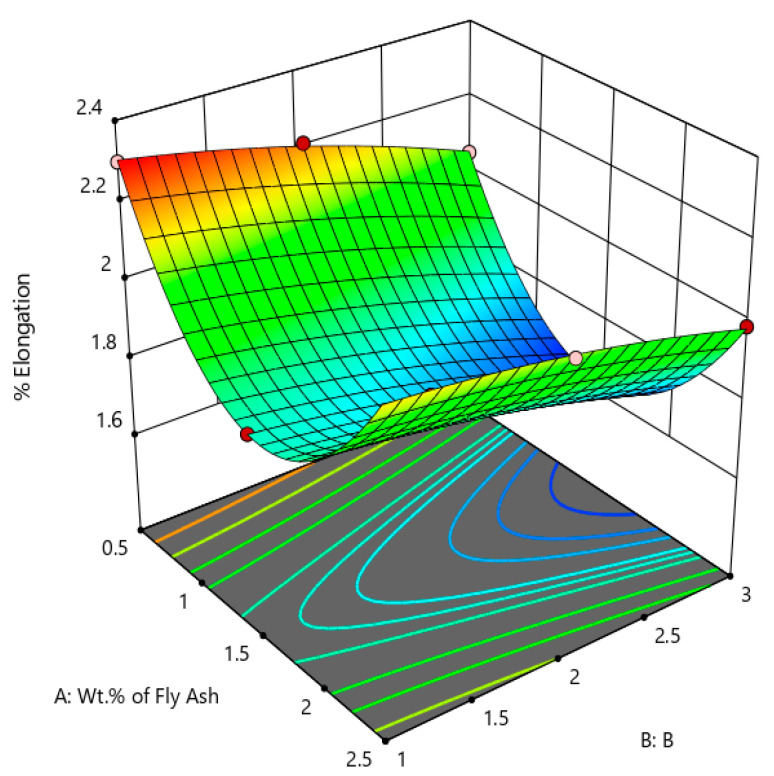
Surface plot and 3D contour plot representing the influence of wt. % and size of fly ash inoculants on % Elongation.

**Figure 8:**
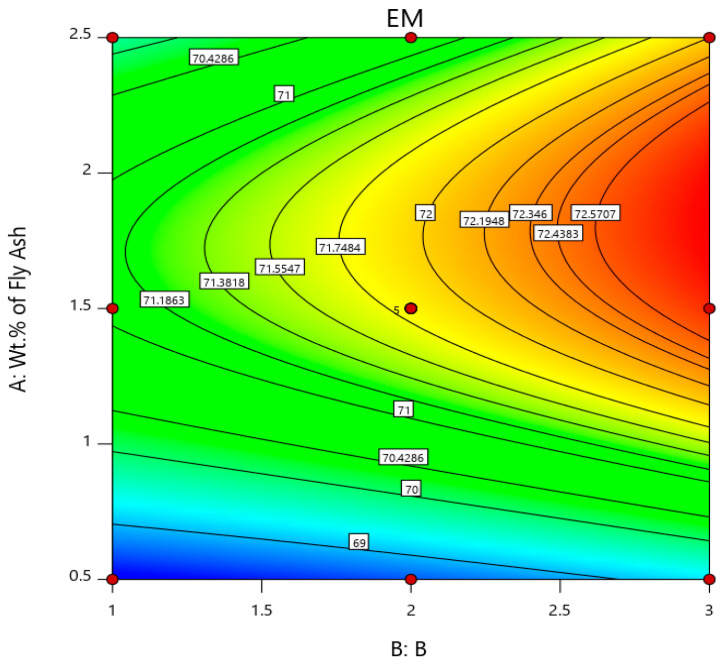

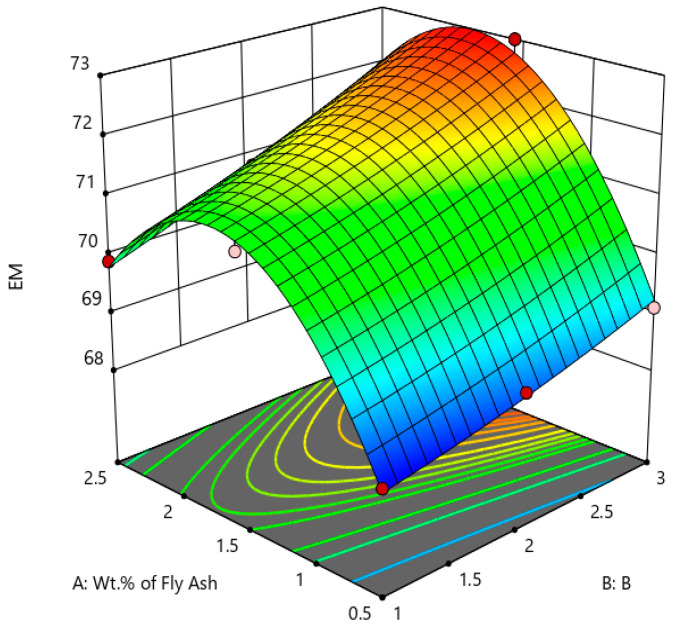
Surface plot and 3D contour plot representing the influence of wt. % and size of fly ash inoculants on Elastic Modulus.

Similarly, the % elongation and elastic modulus plot for the varying weight percentages of fly ash and particle sizes are plotted in Figure 6. It is herewith evident from the plot that the % elongation decreases with the increase in the weight percentage and size range of fly ash flakes, i.e., the % elongation is minimum at 1.55 for 101–125 μm fly ash particles size range for 2 wt. % composition, while it is maximum at 2.3 for 40–75 μm fly ash particles size range for 0.5 wt. % composition, signifying that the composites do not possess enough strength to resist the deformation and undergo subsequent elongation under the influence of the loading before failure. Thus, inoculants are very important to impart the required strength to the composites and improve their elastic (young’s) modulus, which is evident from the graph (Figure 6) and surface and 3D contour plots (Figure 7 and Figure 8), i.e., the elastic modulus is maximum for the composites with 101–125 μm size range fly ash particles for 2 wt. % composition at 73.5 GPa, while the elastic modulus of the composites with 40–75 μm size range of fly ash particles and the composition of 0.5 wt. % is found to be minimum at 68.1 GPa.

It is also determined from the line graph (Figure 6) that the increase in fly ash content beyond 2 wt. % decreases the elastic modulus due to uninhibited deformation of the composites under the sustained loading conditions. Park et al. [30] have reported the interface and synthetic reactions between the atoms and subatomic packings in the matrix and their effect on the characteristics of the composite materials developed. It has been reported from the findings that the interface reaction is accelerated by the grain growth epitaxy due to the atomic reagents in the form of material inoculants.

Gan et al. [31] have carried out substantial work on the friction stir processing of the composites and have found that the process leads to better bonding between the atoms leading to better strength and hardness; especially with respect to the particulates reinforced composites, the findings are in line with the present work, wherein the stir casting process is carried out with the inclusion of inoculants that will eventually trigger the bonding between the Al and Si atoms alongside the peripheral atoms of C leading to increase in strength. However, the Al–Si–C bonds with increased fly ash flake sizes result in embrittlement and reduction in % elongation.

The surface and 3D contour plots (Figure 7 and Figure 8) help in understanding the significance of wt. % and size of fly ash on the ductility viz., % elongation and elastic modulus, and are plotted considering the wt. % of fly ash along (A) abscissa and size of fly ash coded as 1 for 40–75 μm, 2 for 76–100 μm, and 3 for 101–125 μm along (B) Z-axis. From the surface plots and 3D contour plots representing the influence of wt. % and size of the fly ash on the ductility, it is herewith evident that the % elongation is maximum for 0.5 wt. % fly ash and 40–75 μm size range, while it is minimum for 2.5 wt. % fly ash and 101–125 μm size range, owing to embrittlement due to the inclusion of large fly ash flakes that will eventually result in the micro-coring and aggregation due to larger particle sizes and higher wt. % of fly ash flakes, and the elastic strength is maximum for the 1.5 to 2 wt. % interval for the fly ash flakes with size range of 101–125 μm, and this is due to the increase in the stiffness of the composite. It is evident from the surface plots and 3D contour plots that the maximum elongation is for 0.5 wt. % fly ash with size range of 40–75 μm, and it decreases with the increase in the fly ash content and size; however, the stiffness increases.

The findings interpreted from the surface and 3D contour plots are in line with the research findings reported on the effect of inoculation on the properties of the composites. Srivatsan et al. [32] reported that the inclusion of SiC in the AA 2080 matrix phase increased the elastic modulus, yield stress, and strength characteristics; however, % elongation, % reduction of area, and tensile ductility were reduced with increasing reinforcement content. Sahin et al. [33] reported that the UTS of the SiC-coated unidirectional boron fibre-reinforced AA 2014 matrix composites increased with the weight percentage of reinforcements from 169 MPa for the base alloy to 319–530 MPa, due to nucleation and grain growth.

### 3.2. Influence of Inoculation of Fly Ash on Impact Strength

**Figure 9:**
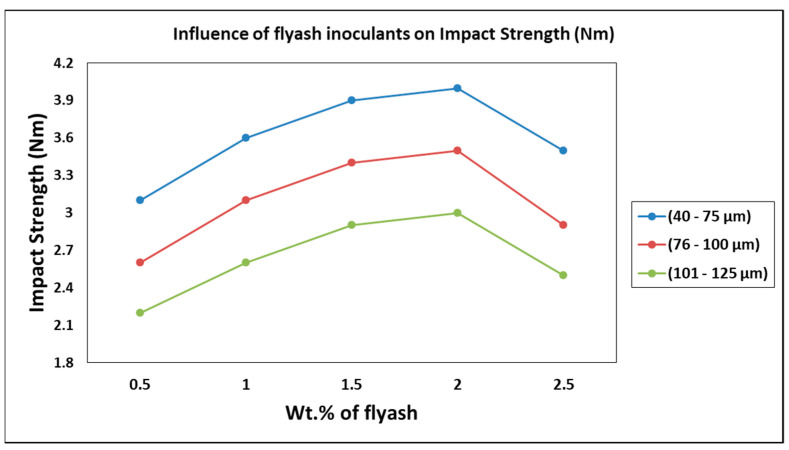
Influence of fly ash inoculants on impact strength.

The influence of inoculation of fly ash on the impact strength is characterized and plotted in Figure 9. It is observed from the graph that the resistance of the composites to impact decreased with the increase in size and wt. % of fly ash, attributed to the embrittlement and coalesce of voids leading to the reduction in impact strength. The composites with fine coalesce of fly ash of 40–75 μm size range as material inoculants have exhibited higher impact strength as compared to the composites with the coarser fly ash (101–125 μm) inoculants. The impact strength decreased by up to 45% in composites with 0.5 wt. % fly ash of 101–125 μm size range as material inoculants as compared to 2 wt. % fly ash of 40–75 μm size range. The impact toughness is found to be low due to the presence of brittle fly ash particles. Singla et al. [34] reported an increase in impact strength with the increase in SiC weight fraction in aluminum matrix up to 15% weight fraction due to coring and micro bonding. Ahlatci et al. [35] reported about the effect of Si in the aluminum matrix and found that the impact toughness increased up to a threshold limit of 8 wt. %, beyond which they have formed agglomerations resulting in the decrease in the impact toughness and, subsequently, the impact strength; this is in parlance with the findings of the present work, where the impact strength increases up to 2 wt. % of fly ash, beyond which the impact toughness decreases leading to a decrease in impact strength due to the micro-coring and aggregation.

**Figure 10:**
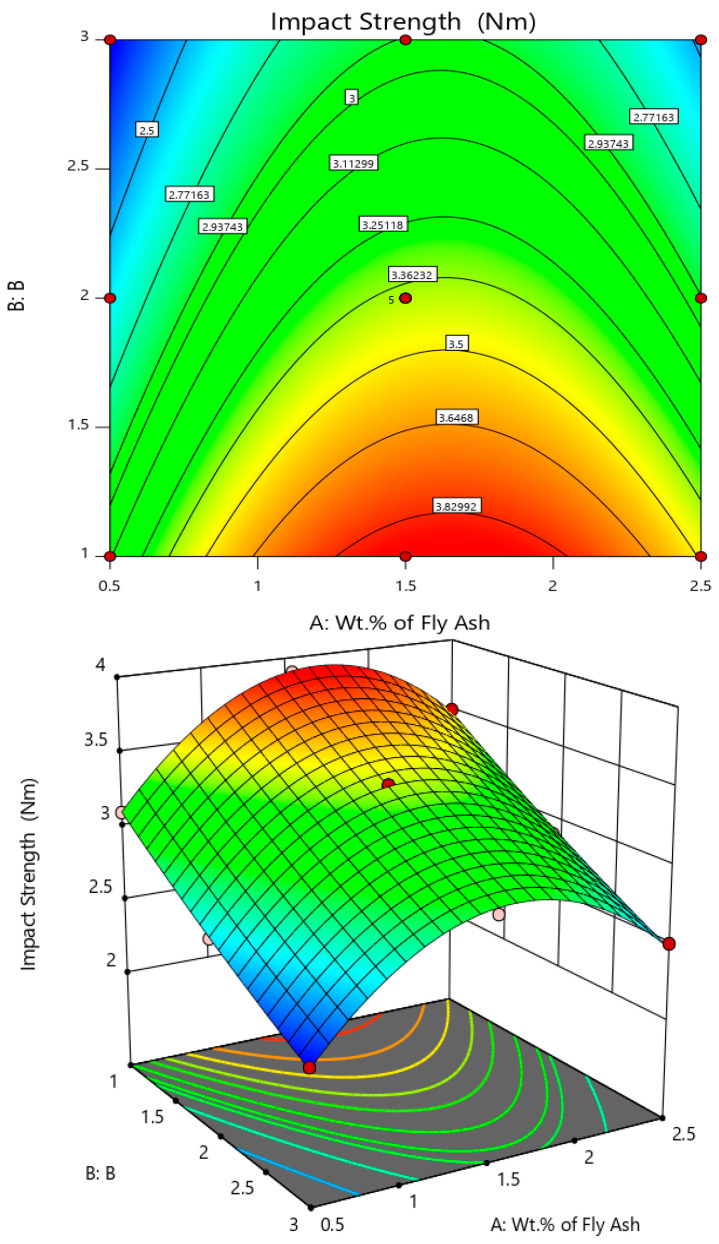
Surface plot and 3D contour plot representing the influence of wt. % and size of fly ash inoculants on Impact Strength.

Further, the variation of impact strength with the varying size range and composition of the fly ash inoculants are interpreted from the surface and 3D contour plot in Figure 10, with wt. % of fly ash along the abscissa (A) and size of fly ash coded as 1 for 40–75 μm, 2 for 76–100 μm and 3 for 101–125 μm along (B) Z-axis. It is herewith noted that the impact strength is maximum for 1.5 wt. % to 2 wt. % interval of fly ash flakes for size range of 40–75 μm.

The findings of the effect of inoculants on the impact strength of the composites are in line with the available research literature. Levashov et al. [36] have emphasized on the need for reinforcements of nano particles in the metal matrix phase to enhance their characteristics, especially with respect to their mechanical and tribological properties, the nano particles added to the metal matrix brings about strong bonding between the atoms due to grain modifications, especially the epitaxial grain growth.

### 3.3. EDS Studies Characterizing the Inoculation of Fly Ash

**Figure 11:**
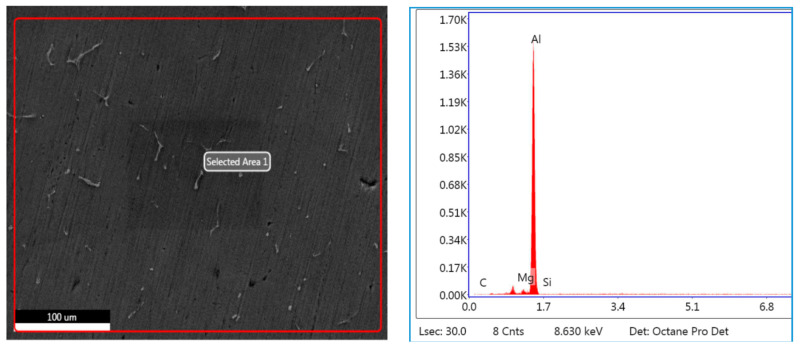
EDS of 40–75 μm, 2 wt. % Fly ash inoculants in AA 5083/7.5SiC.

**Figure 12:**
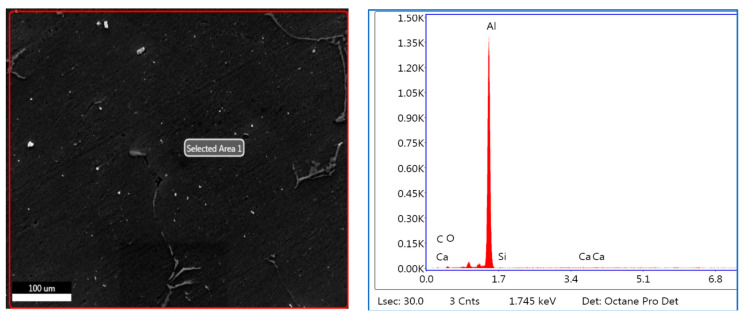
EDS of 76–100 μm, 2 wt. % Fly ash inoculants in AA 5083/7.5SiC.

**Figure 13:**
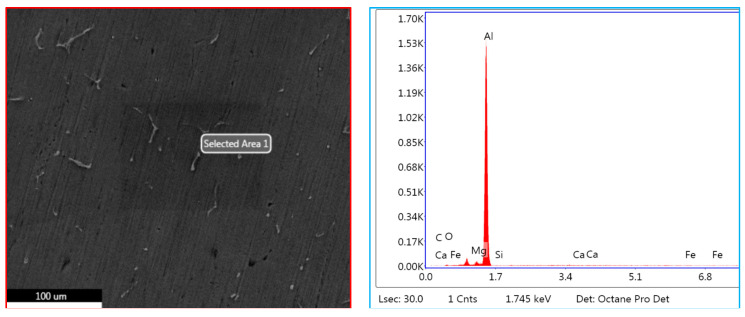
EDS of 101–125 μm, 2 wt. % Fly ash inoculants in AA 5083/7.5SiC.

The EDS studies have been carried out for characterizing the presence of fly ash material inoculants for all the three size ranges, viz., 40–75 μm, 76–100 μm, and 101–125 μm for 2 wt. % composition (Figure 11, Figure 12 and Figure 13); it is herewith evident from the EDS that there is significant presence of Si and C alongside the presence of Al-Si compounds formed due to the inoculation caused by the fly ash particles.

#### 3.3.1. EDS of 40–75 μm, 2 wt. % Fly Ash Inoculants in AA 5083/7.5SiC

The EDS spectrum of 40–75 μm, 2 wt. % Fly ash inoculants in AA 5083/7.5SiC is given in the Figure 11, and the table of the elemental composition is given in Table 3. It is herewith evident that the Al and Al–Si compounds form the maximum elemental compositions in the matrix and the fly ash particles with the size range of 40–75 μm have inoculated to form the Al–Si bonds with the O_2_ element forming the slag with the C_2_Cl_6_ flux, which is skimmed from the top layer of the melt before the molten metal is poured into the metallic dies to get the desired shape and dimensions of the casting.

#### 3.3.2. EDS of 76–100 μm, 2 wt. % Fly Ash Inoculants in AA 5083/7.5SiC

The EDS spectrum of 76–100 μm, 2 wt. % Fly ash inoculants in AA 5083/7.5SiC is given in the Figure 12, and the table of the elemental composition is given in Table 4. It is herewith evident that the Al and C elements form the maximum elemental compositions, along with the O_2_, Al–Si and Ca elements in the matrix and the fly ash particles with the size range of 76–100 μm inoculate to break the bond between the Si–C and form strong Al–Si bond, resulting in better strength as compared to the inoculation of the AA 5083/7.5SiC with 40–75 μm fly ash. Hence, it validates the results that the tensile and yield strength of the composites with 76–100 μm, 2 wt. % inoculants improves with the increase in the size of the fly ash particles.

#### 3.3.3. EDS of 101–125 μm, 2 wt. % Fly Ash Inoculants in AA 5083/7.5SiC

The EDS spectrum of 101–125 μm, 2 wt. % Fly ash inoculants in AA 5083/7.5SiC is given in the Figure 13, and the table of the elemental composition is given in Table 5. It is herewith evident that the Al and O_2_ elements form the maximum elemental compositions, along with the Al–Si, Mg, and traces of Fe and Ca elements in the matrix and the fly ash particles with the size range of 101–125 μm have inoculated in a better way, along with the traces of Fe, Si, and Ca elements with the Al–SiO_2_ bonds resulting in embrittlement with higher strength and lower impact toughness as compared to the inoculation of the AA 5083/7.5SiC with 40–75 μm fly ash. Hence, it validates the results that the tensile and yield strength of the composites with 101–125 μm, 2 wt. % inoculants is the highest and impact toughness is the lowest, resulting in lower impact strength as compared to the fly ash particles with 40–75 μm size range.

The EDS spectrum of all the size ranges ascertain the inoculation in the AA 5083/7.5SiC due to the inclusion of fly ash and the inoculation is maximum for 101–125 μm size range of fly ash particles, and due to the inoculation, the bonding and coalesce of grain occurs resulting in increase in the strength of the composites. However, the ductility and impact toughness of the fly ash particles with large size ranges decrease due to the embrittlement with the formation of oxides and carbides with the aluminum atoms.

### 3.4. X-ray Diffraction Studies

**Figure 14:**
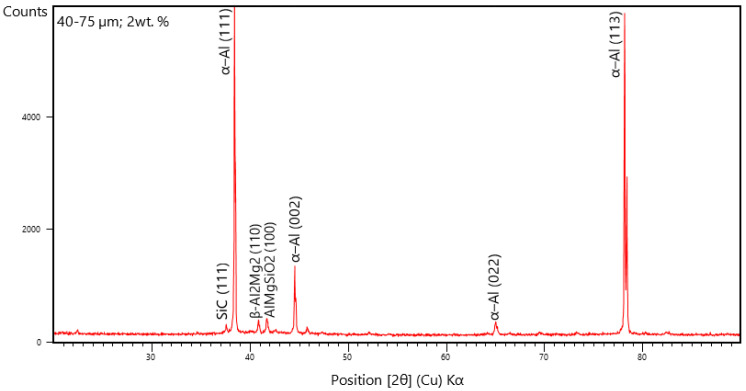
XRD of 40–75 μm, 2 wt. % Fly ash inoculants in AA 5083/7.5SiC.

The XRD diffraction studies are carried out for the phase and grain growth epitaxy of the AA 5083/7.5 SiC composites with fly ash material inoculants. The XRD peaks are derived from the relational ICDD database files for the AA 5083/7.5 SiC composites with 2 wt. % fly ash particles of 40–75 μm as inoculants. It is herewith seen from the XRD, that the peaks correspond to the formation of the α-Al phase, and the aluminum-silicate compounds with oxide formations (Figure 14). Further, from the XRD pattern, the distinct peaks represent the SiC (1 1 1) phase lines represented by the XRD peak formed at a diffraction angle of 37.928°; α-Al (1 1 1) phase lines formed at a diffraction angle of 38.380°, α-Al (0 0 2) phase lines formed at a diffraction angle of 44.612°, α-Al (0 0 2) phase lines formed at a diffraction angle of 64.928°, α-Al (0 2 2) phase lines formed at a diffraction angle of 78.014°, α-Al (1 1 3) phase lines formed at a diffraction angle of 82.204°, and the β-Al_2_Mg_2_ (1 1 0) identified by the 40.992° peaks and AlMgSiO_2_ (1 0 0) compounds represented by the XRD peaks formed at a diffraction angle of 41.876° have ascertained the effect of smaller particulate sizes of Fly Ash particles in forming the α-solid solution of aluminum and the β-solid solution of Al–Mg, which are known to increase the ductility, however decreasing the strength and hardness.

**Figure 15:**
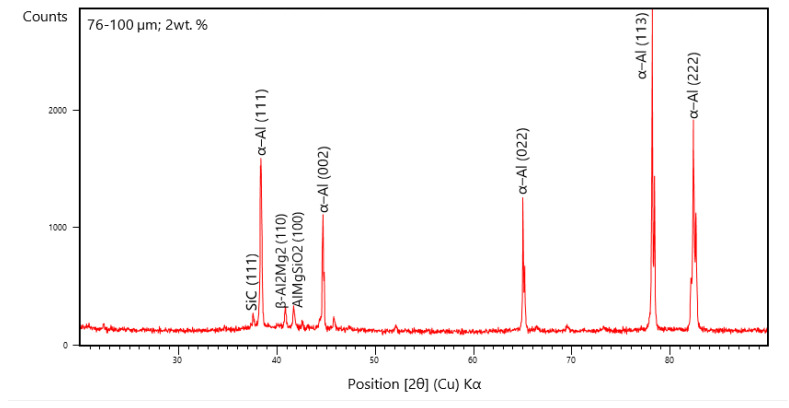
XRD of 76–100 μm, 2 wt. % Fly ash inoculants in AA 5083/7.5SiC.

The XRD peaks are derived from the relational ICDD database files for the AA 5083/7.5 SiC composites with 2 wt. % fly ash particles of 76–100 μm as inoculants (Figure 15). It is herewith seen from the XRD, that the peaks correspond to the formation of the Al–Si–Mg bonds with substantial coring leading to the increase in strength of the composites. Further, from the XRD pattern, the distinct peaks represent the SiC (1 1 1) phase lines represented by the XRD peak formed at a diffraction angle of 37.112°, α-Al (1 1 1) phase lines formed at a diffraction angle of 38.580°, α-Al (0 0 2) phase lines formed at a diffraction angle of 44.724°, α-Al (0 0 2) phase lines formed at a diffraction angle of 65.014°, α-Al (0 2 2) phase lines formed at a diffraction angle of 78.520°, α-Al (1 1 3) phase lines formed at a diffraction angle of 82.312°, α-Al (2 2 2) phase lines formed at a diffraction angle of and the β-Al_2_Mg_2_ (1 1 0) identified by the 41.012° peaks and AlMgSiO_2_ (1 0 0) compounds represented by the XRD peaks formed at a diffraction angle of 42.114° have resulted in slight shift in the α-aluminum and the β-solid solution of Al–Mg lattice parameters, due to the presence of internal stresses, which result in Orowan strengthening and strain hardening at the granular level and subsequent increase in the strength and hardness of the composites with the increase in the size of the fly ash flakes.

**Figure 16:**
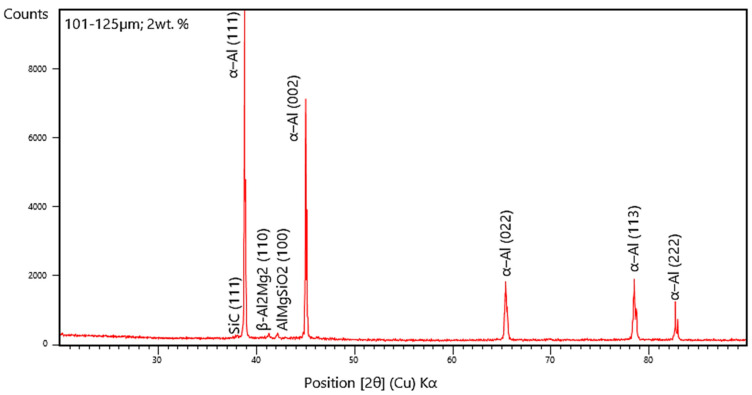
XRD of 101–125 μm, 2 wt. % Fly ash inoculants in AA 5083/7.5SiC.

The XRD peaks are derived from the relational ICDD database files for the AA 5083/7.5 SiC composites with 2 wt. % fly ash particles of 101–125 μm as inoculants (Figure 16). It is herewith seen from the XRD that the distinct peaks are observed for α-Al solid solution along with metal carbide formations, leading to embrittlement and a distinct shift in the phase angle associated with the crystal lattice parameters. Thus, the embrittlement leads to increase in the strength of the composites; however, it decreases the ductility and impact toughness of the composites, majorly due to the hard brittle ceramic formations.

The XRD studies for composites with 101–125 μm size range of fly ash particles in Figure 16, ascertain the grain growth epitaxy and inoculation in the AA 5083/7.5SiC due to the inclusion of fly ash and also represents the peaks due to the formation of compounds of metallic oxides; however, the embrittlement for the 101–125 μm size range of fly ash particles is due to the coalesce of grains resulting in an increase in the strength of the composites, but the formation of oxides and carbides of Al–Mg–Si compounds leads to crystallization of the composites and distinct phases with large intensity counts distinctly identify the coring phenomena and micro-interstitial crystal growth. The peaks corresponding to 39.012° and 45.124°, 66.204°_,_ 78.992°, 83.016° indicate the formation of α-Al solid solution with different lattice parameters, as is evident from the XRD pattern, and the XRD peak corresponding to 41.516° indicate the formation of intermetallic β-phases of Al–Mg, while the peak corresponding to 42.508° indicate the formation of Al–Si–Mg compounds, further from the comparative evaluation of the XRD peaks of the composites inoculated with fly ash particles of 101–125 μm size range with other size ranges, a distinct shift in the phase angles related to crystal lattice parameters are observed due to grain boundary and grain epitaxial changes brought about by the larger fly ash flakes leading to an increase in the strength and hardness due to stronger bonding facilitated by the inoculation brought about by larger size of fly ash flakes (101–125 μm).

From the XRD patterns in Figure 14, Figure 15 and Figure 16, it is evident that the addition of 2% fly ash (ceramics) has significant effect on the structural changes, especially with respect to the Al alloy represented by the distinct peaks in the XRD, particularly the Al_2_Mg_2_ beta phase identified by approximately 41° line and the formation of α-Al solid solution, which is brought about by the inoculation resulting from fly ash dispersions in the matrix phase. Subsequently, the inclusion of large size of fly ash flakes has led to shift in the phase angles related to the crystal lattice parameters due to the presence of internal stresses attributed mainly due to the grain growth epitaxial changes brought about by the increase in the size of the fly ash flakes, resulting in strain hardening and Orowan strengthening leading to increase in strength and hardness of the composites.

### 3.5. Microstructural Studies and EDS Point Analysis

**Figure 17:**
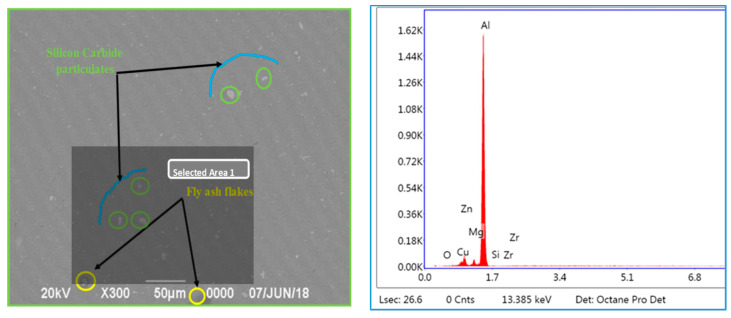
SEM and EDS of 40–75 μm, 2 wt. % Fly ash inoculants in AA 5083/7.5SiC.

**Figure 18:**
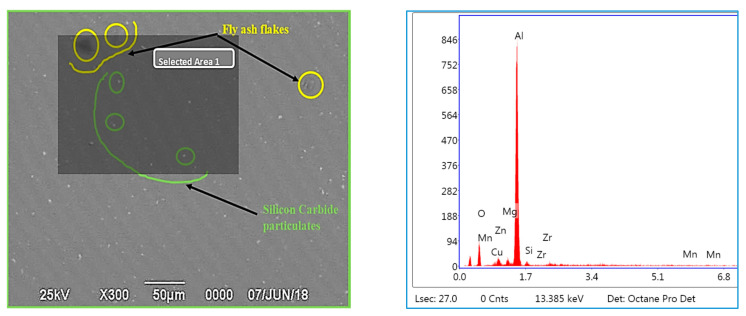
SEM and EDS of 76–100 μm, 2 wt. % Fly ash inoculants in AA 5083/7.5SiC.

**Figure 19:**
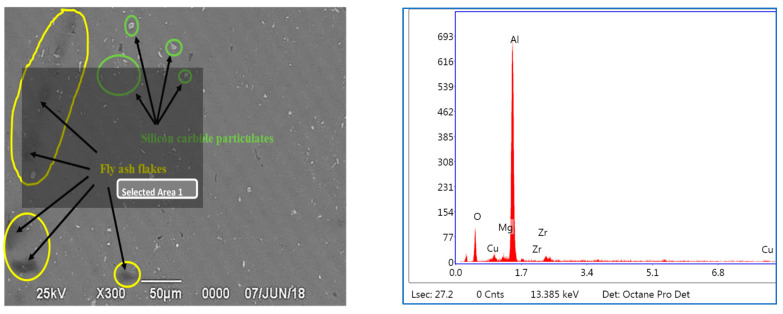
SEM and EDS of 101–125 μm, 2 wt. % Fly ash inoculants in AA 5083/7.5SiC.

The microstructural studies and the EDS point analysis are carried out on the AA 5083/7.5SiC composites with 2 wt. % fly ash particles of varying size ranges of 40–75 μm, 76–100 μm, and 101–125 μm as inoculants (Figure 17, Figure 18 and Figure 19). The SEM images captured at 300× magnification using a Hitachi make SU–8000 series demonstrate that fly-ash flakes comprise precipitator particles. With increment in level of fly ash, there is increment in diffusion of fly ash debris in the composites.

The dispersion of the reinforcements and the inoculants in the aluminum AA 5083/7.5 SiC is a major feature for the characterization of the composite specimens and is vital for the study of the microstructure of the composite materials. It is evident from the microstructural observations and the EDS point analysis for the selected areas in the SEM (Figure 17, Figure 18 and Figure 19) that the increase in size of the fly ash particles in the matrix phase has led to micro-coring and segregation of the inoculant particles in the matrix phase. Further, SEM examination demonstrates that there is a plausibility of atomic reaction between aluminum matrix and fly ash flakes alongside silicon carbide particulates. Moreover, from the SEM, it is evident that the increase in the size range of fly ash material inoculants results in non-uniform scattering in the matrix (Figure 19). Further, the EDS point analysis is used to identify the surface elemental composition and estimate the proportions at various points. It is observed from the Figure 17 that the peaks corresponding to the Al and Si are distinct, followed by the peaks corresponding to the elemental oxygen due to the debonding of oxygen from alumina (Al_2_O_3_) and silicates (SiO_2_) due to inoculation caused by the fly ash particulates. Similarly from the EDS point analysis in Figure 18, it is evident that the Al–Si–O peaks are distinctly evident along with the traces of Mg–Mn–Zn and Cu elements. Consequently, Figure 19 gives an overall mapping of the composite with 101–125 μm, 2 wt. % Fly ash inoculants. The presence of Si, C, O, on the Al surface can be easily identified and also the low atomic number elements of Zn, Zr, and Mg are detected using EDS. Moreover, the XRD diffractograms in Figure 14, Figure 15 and Figure 16 have distinct peaks which are characteristic of Al–Si–Mg–O compounds formed due to the inoculation brought about by the fly ash particles in the matrix phase. These compounds have been identified in the composites with 76–100 µm and 101–125 µm, 2 wt. % fly ash inoculants thereby ascertaining the stronger bonding between the atoms; this is also seen in the EDS of the samples, wherein the distinct elemental peaks corresponding to Al, Si, Mg, and O atoms are also evident, thereby validating the phase identifications corresponding to the peaks in the XRD and more or less the uniform precipitation of SiC brought about by the fly ash inoculants [37,38,39,40].

## 4. Conclusions

The studies on the influence of the use of fly ash as material inoculants in the AA 5083/7.5SiC composites are important to understand the process of inoculation and its impact on the tensile and impact characteristics. The composites with 2 wt. % fly ash inoculants of 101–125 µm size range exhibited higher tensile strength compared to that of the composites (AA 5083/7.5SiC) with 0.5 wt. % fly ash particles, while the ductility and impact strength of the AA 5083/7.5SiC with fly ash inoculants of 40–75 µm size range are higher than those composites with fly ash inoculants of 101–125 µm size range. Further, the increase in weight percentage of fly ash is found to improve the strength characteristics of the composites at the cost of ductility and impact toughness; since the increase in the fly ash content causes embrittlement leading to brittle fracture due to the debonding between the AA 5083-SiC, caused by the inoculation between the atoms, as reported by Boopathi et al. [37], they have investigated the properties of Al-SiC and Al-fly ash composites, fabricated en route stir casting; the studies have resulted in identification of the inoculation capability of fly ash, thereby enhancing the wear and hardness characteristics, along with the tensile and corrosion properties. Babu et al. [38] investigated the improvement of the mechanical properties using the fly ash up to 20% by weight for the aluminum composites synthesized en route stir casting. Mahendra et al. [39] investigated the effect of fly ash content on the mechanical properties, viz., tensile strength, impact strength, compression strength, and hardness, and concluded that the inclusion of fly ash improved these properties due to the inoculation brought about by the grain growth epitaxy in the composite. The EDS has ascertained the findings by distinctly identifying the variation in elemental composition due to inoculation brought about by the fly ash, and also the XRD has identified the phase distinction and grain growth epitaxy due to the inclusion of material inoculants in the form of fly ash. The findings related to the influence of fly ash as inoculants on the dispersion of the reinforcements in composites are also supported by the SEM images, which gives the account of uniformity in distribution of fly ash and SiC reinforcements in the matrix and the extent of dispersion with the varying percentage and size of fly ash particles in the composites. Thus, the composites under the influence of fly ash as inoculants will result in coalescence of voids restricting the movement of dislocations thereby limiting the failure of composites.

## Figures and Tables

**Table 1:** Chemical analysis of the fly ash and its comparison with ASTM C-618 standard.

Chemical Analysis (Composition)	Class C Flyash wt. %	ASTM C-618
SiO_2_	48.2	-
Al_2_O_3_	18.4	-
Fe_2_O_3_	3.7	-
SiO_2_ + Al_2_O_3_ + Fe_2_O_3_	70.3	50 (MIN%)
CaO	19.6	-
f-CaO	5.2	-
CaO + f-CaO	24.8	20 (MIN%)
MgO	1.1	-
SO_3_	1.7	5 (MAX%)
LOI	-	6 (MAX%)
MOISTURE	-	3 (MAX%)

**Table 2:** Chemical composition of the AA 5083 alloy.

Elements in the Alloy	% Present in the Alloy
Cu	0.15
Zn	0.20
Ti	0.20
Cr	0.1
Mg	4.5
Si	0.45
Fe	0.45
Mn	0.7
Al	Remainder

**Table 3:** Elemental Composition from EDS of 40–75 μm, 2 wt. % Fly ash inoculants in AA 5083/7.5SiC

Element	Weight%	Atomic%	Net Int.	Error%	Kratio
AlK	96.37	94.34	722.24	1.99	0.9181

**Table 4:** Elemental Composition from EDS of 76–100 μm, 2 wt. % Fly ash inoculants in AA 5083/7.5SiC.

Element	Weight%	Atomic%	Net Int.	Error%	Kratio
AlK	90.00	82.52	654.39	2.06	0.8451

**Table 5:** Elemental Composition from EDS of 101–125 μm, 2 wt. % Fly ash inoculants in AA 5083/7.5SiC.

Element	Weight%	Atomic%	Net Int.	Error%	Kratio
AlK	93.37	91.29	722.55	2.39	0.8519

## Data Availability

The data presented in this study are available on request from the corresponding authors.
